# Increasing Skin Infections and *Staphylococcus aureus* Complications in Children, England, 1997–2006

**DOI:** 10.3201/eid1603.090809

**Published:** 2010-03

**Authors:** Sonia Saxena, Paula Thompson, Ruthie Birger, Alex Bottle, Nikos Spyridis, Ian Wong, Alan P. Johnson, Ruth Gilbert, Mike Sharland, on behalf of improving Children’s Antibiotic Prescribing Group

**Affiliations:** Imperial College London, London, UK (S. Saxena, R. Birger, A. Bottle); School of Pharmacy, London (P. Thompson, I. Wong); St. George's Hospital National Health Service Trust, London (N. Spyridis); Health Protection Agency, London (A.P. Johnson, M. Sharland); University College London Institute of Child Health, London (R. Gilbert)

**Keywords:** Bacteria, Staphylococcus aureus, children, MRSA, MSSA, primary care, skin infections, England, dispatch

## Abstract

During 1997–2006, general practitioner consultations for skin conditions for children <18 years of age in England increased 19%, from 128.5 to 152.9/1,000 child-years, and antistaphylococcal drug prescription rates increased 64%, from 17.8 to 29.1/1,000 child-years. During the same time period, hospital admissions for *Staphylococcus aureus* infections rose 49% from 53.4 to 79.3/100,000 child-years.

*Staphylococcus aureus* infection is a leading cause of staphylococcal bacteremia in adults (*1*) and children (*2*) in hospitals in the United Kingdom, and recent reports suggest invasive staphylococci are emerging from the community (*3*). Flucloxacillin is the antimicrobial drug recommended for treating *S. aureus* skin infection in UK primary care centers (*4*). Therefore, its use provides a proxy marker of *S. aureus* skin infection in children. Flucloxacillin prescribing in children has increased over the past 15 years (*5*), despite well-documented reductions in prescribing rates for other commonly prescribed antibacterial drugs during 1995–2000 (*6*), which suggests that *S. aureus* skin infections in the community may be increasing.

We examined the incidence of local complications of *S. aureus* disease in children over a 10-year period using nationally representative data from primary care clinicians in England. Ethics approval for this study was obtained from the Independent Scientific and Ethical Advisory Committee, application no. 2006/ISEAC/012.

## The Study

The MediPlus UK database contains anonymized longitudinal data from >500 UK general practitioners who contribute clinical data on >1 million patients (*7*) that have been used widely for research (*8*). Consultations are coded by using the International Classification of Diseases, Tenth Revision (ICD-10), and antimicrobial drug prescriptions are coded by using the British National Formulary for children, Chapters 5.1.1–5.1.3 (*9*). Using Mediplus UK, we extracted data on all skin conditions (ICD-10 code) and atopic dermatitis (ICD-10 code L20) as an index condition in children <18 years of age who saw general practitioners in England from January 1, 1997, through December 31, 2006. We counted prescriptions for all oral and topical antibacterial drugs prescribed for skin infections, and used all oral preparations containing flucloxacillin prescribed for skin conditions as a proxy measure of unresolved *S. aureus* skin infection. We calculated age–sex adjusted annual consulting and prescribing rates by totaling the number of consultations or prescriptions and dividing by the number of person–years contributed by each child in the registered population for each calendar year. We then directly standardized these rates by using the age–sex distribution for the reference year 2000.

The Hospital Episode Statistics (HES) database has recorded all inpatient hospital activity in National Health Service hospitals across England since 1989 and is used widely to monitor disease trends in England (www.hesonline.nhs.uk) (*10*). The main reason for admission, i.e., primary diagnosis, is recorded by using ICD-10 codes. We used HES data to calculate age–sex adjusted admission rates per 100,000 resident population for children <18 years of age for each calendar year from January 1, 1997, through December 31, 2006, for conditions commonly caused by *S. aureus*, including septic arthritis (ICD-10 codes M00.0 for staphylococcal arthritis and M00.9 for pyogenic arthritis), osteomyelitis (M86), and locally invasive skin infections (L02, cutaneous abscesses and boils; L03, cellulitis). Rates were calculated as the total number of admissions per year divided by the mid-year estimate of the number of children residing in England (using the 2000 population in England as the reference population) (*11*). Confidence intervals (CIs) were generated with a Poisson approximation. We used linear regression to test for linear trends in age–sex adjusted admission rates across the period. We used Stata version 9 software (Stata Corp., College Park, TX, USA) for all statistical analysis.

**Figure 1:**
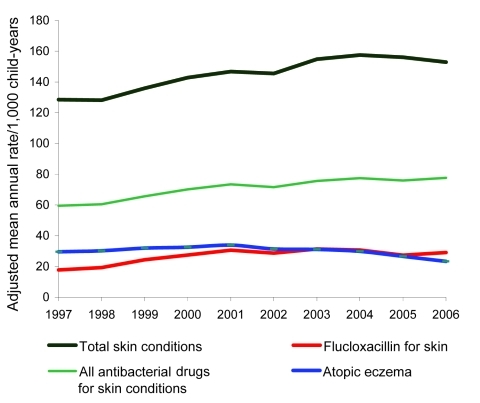
General practitioner consultation and prescribing rates for all skin conditions in children <18 years of age, England, 1997–2006.

The Mediplus database contained 2,821,372 child-years of follow-up during 1997–2006. General practitioner consultation rates for all skin conditions in children rose from 128.5 (95% CI 127.2– 129.8) per 1,000 child-years to 152.9 (95% CI 151.4–154.5) per 1,000 child years (p = 0.011). Atopic eczema consultation rates decreased during this time (Figure 1).

**Figure 2:**
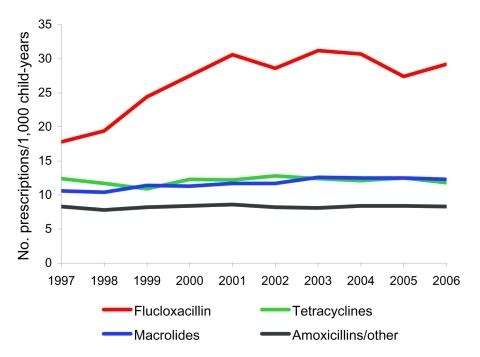
Prescribing rates for antibacterial drugs for children <18 years of age, England, 1997–2006.

In parallel with the rising number of skin consultations was a 64% increase in prescribing rates for antistaphylococcal drugs (flucloxacillin), from 17.8 (95% CI 17.3–18.3) to 29.1 (95% CI 28.5–29.8) prescriptions per 1,000 child-years (p<0.001) (Figure 2). Prescribing of all other antibacterial drugs for children for any reason decreased from 541.4 (95% CI 538.8–544.1) per 1,000 child-years to 484.3 (95% CI 481.6–487.0) per 1,000 child-years (Table 1). Flucloxacillin was the most commonly prescribed antibacterial drug for all skin conditions (37%). Prescribing rates for other classes of antibacterial drugs used for skin infections, notably, combined preparations of amoxicillin and clavulanic acid 2% and fusidic acid (<2%), were stable over the time period (Figure 2).

**Table 1:** Age- and sex-adjusted skin condition consultation and antibacterial drug prescribing rates for children <18 years of age, England, 1997–2006 *

Year	Rate/1,000 child-years† (95% CI)				
	GP consultations for skin conditions	Antibacterial drugs for skin conditions	Flucloxacillin for skin conditions	All flucloxacillin	All antibacterial drugs
1997	128.52 (127.21–129.83)	59.53 (58.63–60.43)	17.76 (17.28–18.25)	35.04 (34.36–35.72)	541.42 (538.75–544.09)
1998	128.17 (126.87–129.47)	60.44 (59.54–61.34)	19.33 (18.83–19.84)	37.35 (36.65–38.05)	498.64 (496.09–501.20)
1999	135.91 (134.57–137.24)	65.68 (64.74–66.61)	24.39 (23.83–24.96)	44.03 (43.27–44.79)	438.24 (435.84–440.63)
2000	142.80 (141.44–144.17)	70.17 (69.21–71.13)	27.46 (26.86–28.06)	48.89 (48.09–49.69)	431.80 (429.42–434.18)
2001	146.75 (145.36–148.14)	73.43 (72.45–74.41)	30.65 (30.01–31.28)	52.76 (51.93–53.60)	460.68 (458.21–463.14)
2002	145.56 (144.17–146.94)	71.64 (70.68–72.61)	28.70 (28.09–29.32)	51.39 (50.56–52.21)	443.77 (441.34–446.19)
2003	154.81 (153.37–156.24)	75.69 (74.69–76.68)	31.39 (30.74–32.04)	56.00 (55.13–56.87)	459.94 (457.45–462.43)
2004	157.56 (156.07–159.05)	77.51 (76.47–78.55)	30.75 (30.09–31.42)	56.22 (55.32–57.12)	458.60 (456.04–461.16)
2005	156.05 (154.54–157.57)	75.98 (74.93–77.04)	27.32 (26.68–27.96)	53.58 (52.68–54.47)	489.24 (486.55–491.93)
2006	152.94 (151.44–154.45)	77.62 (76.54–78.69)	29.13 (28.47–29.80)	56.07 (55.15–56.99)	484.30 (481.61–486.98)
p value‡	0.011	0.005	0.001	0.001	0.005

*Data from International Marketing Systems Health. CI, confidence interval; GP, general practitioner. †Directly age-sex adjusted to 2000 sample population. ‡p value test for linear trend across years.

During 1997–2006, unplanned hospital admission rates for skin, bone, and joint infections in all children increased by 49% from 53.4 (95% CI 52.1–54.7) to 79.3 (95% CI 77.7–80.9) per 100,000 child-years (p<0.001) including cellulitis (67.8% increase; p<0.001), skin abscesses (36.7% increase; p <0.001), and osteomyelitis (46.1% increase; p = 0.004) (Table 2). This trend was consistent across all age groups. Admission rates for septic arthritis increased but the result of the test for trend was not significant (p = 0.128).

**Table 2:** Age- and sex-adjusted hospital admission rates for skin, bone, and joint infections in children <18 years of age, England, 1997–2006*

Year	Rate/100,000 child-years (95% CI)				
	All skin, bone, and joint infections	Cutaneous abscesses and boils	Cellulitis	Osteomyelitis	Septic arthritis
1997	53.38 (52.07–54.70)	25.19 (24.29–26.10)	19.81 (19.01–20.61)	4.81 (4.42–5.21)	3.57 (3.23–3.91)
1998	57.99 (56.62–59.36)	27.37 (26.43–28.32)	22.24 (21.39–23.09)	4.82 (4.43–5.22)	3.55 (3.21–3.89)
1999	61.34 (59.93–62.75)	28.86 (27.89–29.82)	23.63 (22.75–24.50)	5.24 (4.82–5.65)	3.62 (3.28–3.96)
2000	64.95 (63.49–66.41)	28.94 (27.97–29.91)	26.48 (25.55–27.41)	5.70 (5.27–6.14)	3.83 (3.47–4.18)
2001	61.99 (60.56–63.41)	28.02 (27.06–28.98)	25.25 (24.34–26.16)	5.55 (5.12–5.97)	3.17 (2.85–3.50)
2002	63.65 (62.20–65.09)	29.65 (28.66–30.64)	25.01 (24.11–25.92)	5.45 (5.02–5.87)	3.54 (3.19–3.88)
2003	71.15 (69.62–72.68)	32.71 (31.68–33.75)	29.26 (28.27–30.24)	5.39 (4.97–5.81)	3.79 (3.43–4.14)
2004	73.66 (72.11–75.22)	32.98 (31.94–34.02)	30.63 (29.63–31.64)	6.11 (5.66–6.56)	3.94 (3.58–4.30)
2005	76.26 (74.67–77.84)	35.17 (34.10–36.25)	32.03 (31.00–33.05)	5.63 (5.20–6.07)	3.43 (3.09–3.76)
2006	79.28 (77.66–80.89)	34.43 (33.37–35.49)	33.23 (32.18–34.28)	7.04 (6.55–7.52)	4.58 (4.19–4.97)
p value†	<0.001	<0.001	<0.001	0.004	0.128

*Data from Hospital Episode Statistics. Rates directly adjusted to mid-year 2000 resident population for England. International Classification of Diseases, Tenth Revision, codes for primary diagnosis at admission: cutaneous abscesses and boils, L02; cellulitis, L03; osteomyelitis, M86; septic arthritis (staphylococcal arthritis, M00.0, or pyogenic arthritis, M00.9). All of these codes were included in the category “all skin, bone, and joint infections.” CI, confidence interval. †p value for test for linear trend across years.

## Conclusions

The increasing incidence of childhood skin infections and prescribing of the major antistaphylococcal drug flucloxacillin seen in UK primary care, coupled with concurrent increases in childhood hospital admissions for skin bone and joint infections caused by *S. aureus* in hospitals in England, suggests an increase in community-onset *S. aureus* disease in England over the past 10 years. The time frame, a large nationally representative population, use of prospectively collected data, and consistency of patterns make it unlikely our findings arose by chance. Because HES data records primary diagnosis when patients are admitted, most infections will be community-, not hospital-, acquired. Flucloxacillin has remained the treatment of choice for *S. aureus* skin infections in UK primary care for decades (*9*), and its increased use is not explained by treatment drift from other groups of antibacterial drugs used to treat skin infections or by increasing antibacterial drug treatment of atopic eczema colonized with staphylococci. Total unplanned admission rates in children <19 years of age have increased by 13% during 1997–2006 (*12*), but these increases are modest compared with the 49% increase in *S. aureus* complications seen over the same time frame in our study.

Limitations of our study include the use of clinically coded proxy measures for *S. aureus* infections that are subject to recording bias. Because of the lack of microbiologic surveillance, we could not differentiate whether increases in *S. aureus* disease were caused by methicillin-sensitive *S. aureus* or methicillin-resistant *S. aureus.* Antimicrobial drugs for skin infections are available only by prescription in the UK but not exclusively from GPs. Thus, our prescribing data excluded prescriptions issued from other healthcare settings. Our findings that admission rates for osteomyelitis, boils, and cellulitis increased but septic arthritis rates were stable might be because septic arthritis is also caused by pneumococci, β-hemolytic streptococci, and gram negative organisms (*13*).

A growing body of evidence supports our findings of increases in community-onset *S. aureus* disease in children. Hospitalizations for *S. aureus* disease in all age groups are increasing; in several countries severe skin infections, particularly among children, are rising, caused by strains of *S. aureus* producing the Panton-Valentine leukocidin (*14**,**15*). Although currently no formal surveillance of this strain in the UK is available, referrals of isolates of *S. aureus* positive for Panton-Valentine leukocidin to the national Staphylococcal Reference Unit increased each year from 224 in 2005 to 1,361 2007. What is not known is whether *S. aureus* community-acquired infections in children have added to the recently reported increases of *S. aureus* infection and bacteremias acquired in hospital settings (*4*). Further work is required to monitor *S. aureus* disease and antimicrobial drug resistance and to identify community risk factors for *S. aureus* disease in children.
